# 

**Published:** 1825-11

**Authors:** 


### MONTHLY CATALOGUE OF MEDICAL BOOKS.

⁂ *It having been hinted to us that gentlemen, sending copies of their Works, prefer having their titles given at length, it is our intention, in future, to comply with this suggestion: but it is to be observed, that no books can be entered on this List except those sent to us for the purpose;—finding, in the lists hitherto transmitted, that the names of works have frequently been given as published, which have not appeared for weeks, or even months, after.*

Lectures and Observations in Medicine. By the late Matthew Baillie, M.D. (Printed, but not published.)—London, 1825.

The Works of Matthew Baillie, M.D. To which is prefixed, an Account of his Life, collected from authentic Sources, by James Wardrop, Surgeon Extraordinary to the King, &c. &c. &c. In two volumes.—London, 1825.

On the Removal of Stone from the Bladder, without the Use of Cutting Instruments: containing a general Review of the Subject; together with a Description and Plates of the Instruments invented by Dr. Civiale and others, and Details as to the Mode of using them. By H. G. Belinaye, Esq.—London, 1825.

A Century of Surgeons on Gonorrhœa, and on Strictures of the Urethra.—London, 1825.

An Introduction to the Use of the Stethoscope; with its Application to the Diagnosis in Diseases of the Thoracic Viscera; including the Pathology of these various Affections. By William Stokes, M.D.—London, 1825.

A Course of Dissections, for the Use of Students. By Herbert Mayo, Surgeon, and Lecturer on Anatomy.—London, 1825.

☞ We recommend this volume to Students desirous of prosecuting their labours in the dissecting-room with advantage.

An Address to the Inhabitants of Lancashire, and of the adjoining Counties, on the present State of the Medical Profession; with Remarks on the Elementary Education of the Student, and the best Means of its Acquirement. Intended to shew the Practicability and Importance of establishing a School (on a more extended Scale) in Manchester, for the Cultivation of Medical and Surgical Knowledge. By Thomas Turner, Member of the Royal College of Surgeons, London; Lecturer on Anatomy, &c. &c. &c.—London, 1825.

### NOTICE TO CORRESPONDENTS.

*We have received* "*An Essay on the Natural, Chemical, and Medical History of Water, in its simple and combined States; including an Account of the Chemical Composition and Medical Effects of the principal Mineral Waters in the United Kingdom of Great Britain and Ireland, and also of the Continent of Europe.* By Michael Ryan, M.D. Member of the Royal College of Surgeons, London, &c. &c. &c." *We regret that it was not in our power to comply with the wishes of the author, by publishing it in the present Number; but we have given the title in full, as he requested.*

*We have likewise received the Papers of* Mr. Fowkes, Dr. Torrie, Dr. Villers, Mr. Hobart, Mr. Sym, Dr. Fox, Mr. Richmond, Dr. Granville, Dr. Stoker, Dr. Gregory, *and* Dr. Whiting.

